# A Striking Response to Lithium in Kleine–Levin Syndrome

**DOI:** 10.3389/fneur.2014.00033

**Published:** 2014-03-21

**Authors:** Olafur Sveinsson

**Affiliations:** ^1^Department of Neurology, Karolinska University Hospital, Stockholm, Sweden

**Keywords:** Kleine–Levin syndrome, lithium, hypersomnia, cognitive disorders, hypothalamus

## Abstract

Kleine–Levin syndrome (KLS) is an episodic hypersomnia with cognitive disturbances such as confusion, apathy, and derealization. Hyperphagia and hypersexuality occur in around 50% of cases. No evidence-based treatments have been established for KLS. Many drugs have been tried, most often with little success. Here, a case with a striking response to lithium is presented.

## Introduction

Kleine–Levin syndrome (KLS) is classified as a periodic hypersomnia. Diagnostic criteria have been put forward (Table 1) (1). Episodic hypersomnia and cognitive disturbances constitute the core abnormalities. Although hyperphagia and hypersexuality were originally thought to be the cardinal symptoms of KLS, they are only present in around 50% of cases. Recent studies have suggested that recurrent hypersomnia and at least one of the following symptoms: confusion, apathy, or derealization, constitute improved criteria (Table 1).

**Table 1:** **Diagnostic criteria for recurrent hypersomnia and Kleine–Levin syndrome (1)**.

∙ Patient experiences recurrent episodes of excessive sleepiness lasting for 2 days to 4 weeks
∙ Episodes recur at least once per year
∙ Alertness, cognitive function, and behavior are normal between episodes and hypersomnia is not better explained by another sleep, medical, neurological, mental disorder, medication use, or substance abuse
In addition to all recurrent hypersomnia criteria, the patient should also have at least one of the following
∙ Cognitive abnormalities – e.g., derealization, confusion, and hallucinations
∙ Abnormal behavior – e.g., irritability, aggression, or uncharacteristic behavior
∙ Hyperphagia
∙ Hypersexuality

Unfortunately no evidence-based treatments have been established for KLS. Many drugs have been tried, mostly with little success. Possibly lithium has shown the most effect or around 40% (2). Here, a case with a dramatic response to lithium is presented. Why lithium might have an effect in this treatment resistant disorder is briefly discussed.

## Case

A former healthy 19-year-old male began suffering from 1 to 2-week long episodes (with regular 4–5 week intervals) where he was without any energy and could not attend school or do his daily routines. During the episodes he had difficulty in concentrating, slowness of thought, decreased short time memory, reduced physical energy, and feeling of derealization. He described himself being like a zombie or in an unreal state. The patient was examined by a psychiatrist both during and between the episodes, which did not reveal any psychiatric disorder. There was no history of drug abuse.

Besides sleeping during the episodes he could at best watch a simple television program. He became extremely irritable when his parents pressed him to do anything. He spent most of his time in his bedroom and slept for 14–20 h/day for 1–2 weeks. During the episodes, he preferred to be in the dark and avoided communication with other people, even his best friends. Interestingly, he had almost no memory of the episodes. Between the episodes, he was perfectly normal, attending school without difficulty, and playing golf at a high level. During the episodes, his parents had to wake him up to give him food. He went self to the bathroom, but otherwise he stayed in bed. The attacks started relatively quickly, under a few hours and resided under a few hours. During the attacks he did not eat excessively and there were no signs of hypersexuality. No relative has suffered from a similar condition. Extensive psychiatric examination did not reveal any signs of psychiatric disease or depression. There were no signs of epilepsy. Somatic and neurologic examination was normal. Magnetic resonance imaging was normal. Extensive blood tests were normal. EEG showed some hypoactivity in the frontal-lobes, but no signs of epilepsy. At first, lamotrigine was titrated up to 100 mg twice daily and maintained for a few months without any effect. After 2 years his treatment was changed to lithium and serum levels kept between 0.6 and 0.9 mmol/l. Since then he has not had an attack for 3 years. He tolerates the medication well and has not wanted to stop the treatment.

## Discussion

Kleine–Levin syndrome is considered a rare disorder (no population-based studies are available). The cardinal symptom in KLS is recurrent episodes of hypersomnia. Other symptoms such as cognitive affection, hyperphagia, and hypersexuality are observed in various degrees (1). The disorder is somewhat more common in males (63%) and starts during adolescence and young adulthood (2). The cause of KLS is unknown. Intermittent hypothalamic dysfunction seems likely, but there is only limited evidence for this. Postmortem studies are rare because the condition is not fatal. Microglial infiltrations in the thalamus and midbrian as well as decreased pigmentation in the substantia nigra have been described (3).

Hypersomnia, which is the cardinal symptom of the disorder, is compulsory for diagnosis. An exemplary episode starts relatively quickly within a few hours, where the observers see the patient become increasingly tired, often after a trigger such as infection alcohol intake or head trauma (3). During the episodes, the patient usually sleeps between 12 and 24 h/day. The patient in the case above did not have symptoms of hypersexuality/hyperphagia. Possibly too much weight has been laid on those symptoms, conceivably leading to under diagnosis of KLS. On the other hand, cognitive difficulties such as memory defects, confusion, attention, and concentration are always present. Patients describe a state of mental viscosity/slowness and derealization or feeling “strange,” “detached,” or “different” is very common. As in our patient, many patients have anterograde amnesia of the episodes.

In all cases, brain computerized tomography and magnetic resonance imaging are normal. Studies performed in the midst and between the episodes with SPECT, PET, and perfusion MRI have shown hypoperfusion in the frontal-lobe, temporal-lobe, and hypothalamic areas (3). On the one hand, cognitive disturbances and derealization could be explained by temporal-lobe dysfunction. On the other hand, apathy and disinhibition could be explained by frontal-lobe dysfunction. Thalamus and hypothalamus involvement, which have been frequent findings, could explain hypersomnia or apparent sleeplike behavior. Interestingly, hypoperfusion has been shown to be present in 50% of the patients in the temporal-lobe during asymptomatic periods (3). Hypoperfusion in the frontal-lobe and the basal ganglia has also been found (3). This suggests that certain subclinical abnormalities are present between the attacks. This also leads to the question of abnormalities after KLS has gone into remission. A low-frequency high-amplitude wave (delta or theta) mainly in the bilateral temporal or temporofrontal area and a non-specific frontal slowing of background activity is often seen on the EEG. This parallels the SPECT findings.

Many medications have been tried in KLS, most without success. Stimulants have been tested. Amphetamines significantly reduce sleepiness in patients, but unfortunately they do not improve and can even make the more troublesome behavioral and cognitive disturbances worse. In the largest review on KLS (108 patients) only lithium showed an effect significantly higher than no treatment (OR 3.8, *P* = 0.02) (2).

Lithium had a dramatic effect on the patient above. Interestingly, lithium has also an effect on cluster headache, a periodic disorder that might have its origin in the hypothalamus, like KLS. Lithium is most known for its effect in bipolar disorder, which is a disorder of periodicity, like KLS. Lithium up-regulates glutamate reuptake. Hence, it decreases glutamate availability at synapses. This might have an anti-manic effect since gluatmate is an excitatory neurotransmitter. It is of course unknown, if through this mechanism it exerts its effect on KLS (4).

Even today, little is known about lithium’s mechanism of action despite being known and used for over 40 years in bipolar disorders. The drug has a number of hypothetical mechanisms, which will not be elaborated here, but it induces a shortage of inositol in the brain. This causes a deficiency of substrate for phosphatidylinositol resynthesis, but solely in overactive neurons (5), which might clarify lithium’s effect with minimal effects on normal behavior (6).

Firstly, since the patient did not have symptoms of overeating or hypersexuality one can only claim that lithium had a striking effect on the recurrence of hypersomnia and this possible therapeutic effect should be investigated more deeply. Secondly, KLS, cluster headache, and bipolar disorders, all seem to respond to lithium? This should be looked in to more closely. Thirdly, on the cellular level, what is the reason for lithium’s observed effect in KLS? Is it through a shortage of inositiol with a secondary possible dampening on overactive neurons or is it through decreased glutamate availability at synapses or is there an alternative explanation. This should be studied further. After observing this case and taking into account larger case series, lithium should without a doubt be tried as a therapeutic option for KLS.

## Conflict of Interest Statement

The author declares that the research was conducted in the absence of any commercial or financial relationships that could be construed as a potential conflict of interest.
